# The impact of gender on the self-confidence of practical and surgical skills among OBGYN residents: a trinational survey

**DOI:** 10.1007/s00404-023-07202-6

**Published:** 2023-09-11

**Authors:** Tara Meister, Philipp Foessleitner, Georg Breuer, Franziska M. Winder, Martine Favero, Margareta Friemann, Benedict Krischer, Martin Weiss, Karin Windsperger

**Affiliations:** 1 Division of Obstetrics and Feto-Maternal Medicine, Department of Obstetrics and Gynecology, Medical University of Vienna, Währinger Guertel 18–20, 1090 Vienna, Austria; 2 Department of Obstetrics and Gynecology, University Hospital Tulln, Tulln, Austria; 3 Frauenklinik, Kantonsspital St., Gallen, Switzerland; 4 Rhypraxis, Feuerthalen, Switzerland; 5 Department of Gynaecology and Obstetrics, Municipal Clinical Center Lüneburg, Lüneburg, Germany; 6 Frauenklinik Spital Zollikerberg, Zurich, Switzerland; 7 Department of Women’s Health, Eberhard Karls University, 72076 Tübingen, Germany

**Keywords:** Obstetrics, Gynecology, Residency, Education, Surgery, Gender disparities, Self-confidence

## Abstract

**Introduction:**

Gender disparities exist in the OBGYN discipline. This study investigates, for the first time, whether gender impacts on the confidence of practical and surgical skills among OBGYN residents, and of being prepared to work as a specialist.

**Methods:**

The gynecological societies of Austria, Germany, and Switzerland established a web-based survey of 30 questions that was sent to all registered OBGYN members-in-training from August to September 2020. Data collection, controlling and analysis were performed by the Swiss Federal Institute of Technology in Zurich (ETH).

**Results:**

A total of 422 participants took part in the survey, of which 375 (88.9%) were female, 46 (10.9%) were male, and one (0.2%) was divers. The diverse participant was excluded from further analyses. The gender distribution was comparable in all three countries. Multiple regression analyses showed that gender is an independent variable significantly impacting on the confidence levels in performing standard gynaecological (*p* = 0.03) and obstetric (*p* < 0.001) procedures. Similarly, the feeling of confidence in being prepared for working as a specialist in a clinic showed to be gender-dependent (*p* < 0.001), however, not the feeling of being prepared for working as specialist in an outpatient setting (*p* = 0.37). The “female factor” significantly decreases the confidence rating for surgical and practical skills and for working in a hospital. Covariates including year of training, country, workload, receiving regular feedback, and implemented simulation training were included in all analyses.

**Discussion:**

Improvements of residency programs to promote female doctors to overcome factors reducing their confidence in their own OBGYN skills are highly warranted.

## What does this study add to the clinical work

**Table:** 

Gender disparities exist in the self-confidence of practical/surgical skills among OBGYN residents. Improvements of residency programs to promote female doctors are highly warranted.	

## Introduction

The number of women choosing medicine as a career has steadily increased in recent decades, leading to significant shifts in the gender composition of medical students and the physician workforce in many countries [1–3]. Despite this phenomenon—known as the “feminization of medicine”—women are still underrepresented in the higher echelons of medicine. Female doctors are less likely to pursue a hospital or an academic career and only 16% of medical directors are female [4, 5]. The field of obstetrics and gynaecology (OBGYN) is no exception in this regard [6].

The first officially recognized and practicing female gynecologist in Germany was Hermine Heusler-Edenhuizen, who obtained her OBGYN specialty certification in 1909. Being the only woman at her university, she had to ask the professors for permission to attend a lecture and had to prove her physical ability to perform obstetric procedures [7]. Heusler-Edenhuizen was an important pioneer who significantly paved the way for women entering medicine. However, even today there are still multiple obstacles female doctors must face when pursuing a career in OBGYN.

Female doctors are still primarily responsible for childcare, and thus they work more often part-time than their male colleagues [8]. Their demands for reduced working hours and the provision of childcare facilities, however, are seldomly met, which makes juggling work and family life difficult [8]. When it comes to pursuing a medical career, working part-time, however, diminishes the chances of professional success [9]. Furthermore, studies showed that women usually struggle to find a mentor and/or same-sex role models [10, 11]. From the field of general surgery, it is also known that female doctors perform surgical procedures less often compared to their male colleagues, although they show equal or better skills improvement following simulation trainings [12, 13].

Although previous studies have found gender disparities in medical disciplines, it has not yet been investigated whether gender impacts on the self-confidence of surgical and practical skills among OBGYN residents. For the first time, this study analysed whether OBGYN training shows gender-related differences in terms of feeling secure in performing standard surgical and obstetric procedures, as well as in being prepared to work as a specialist.

## Materials and methods

### Study design

From 1st August till 30th September 2020 an online survey was performed by the Austrian, German, and Swiss gynecological societies. A web-based questionnaire entitled "The quality of postgraduate training in OBGYN in Germany, Austria and Switzerland” was e-mailed together with an invitation letter to registered members-in-training of the societies. The participants were enrolled anonymously and multiple participations by one person were excluded by an anonymous IP address check. The survey was circulated and controlled by the Swiss Federal Institute of Technology in Zurich (ETH), Department for Health Sciences and Technology, Consumer Behavior. After four reminders, 422 members in total responded to the survey, including 97 Austrian OBGYN residents (23%), 209 German OBGYN residents (49.5%), and 116 Swiss OBGYN residents (27.5%). This corresponded to a response rate, in dependence to the countries, of up to 55%.

### Questionnaire

The digital questionnaire was designed by the representatives of the trainee networks of the OEGGG, DGGG, and SGGG in German language and contained 30 questions. These covered demographic factors, current training situation, workload, theoretical training content, practical and surgical skills, daily task division, mentoring and feedback culture, evaluation of training progress and logbook, and training satisfaction. As it is mandatory to take night shifts within the residency training, and the European Working Time Directive is implemented in the countries Austria, Germany, and Switzerland we have not evaluated this information in detail [14]. The format of the answers varied; some were open and others had to be selected according to a 7-point Likert scale, ranging from “does not apply at all” to “does fully apply”. The survey was described in detail in Winder et al. [15]. For validation, a pre-test on 30 residents (10 from each country) was carried out prior to the main study. Following inconsistent responses and critical feedback, some of the questions were rephrased. Data collection, control, and processing for analysis was carried out by the ETH.

### Statistical evaluation

The statistical analysis was carried out using IBM SPSS Statistics 27 software. Data were presented as frequencies (*n*) and proportions (%), as medians with minimum and maximum levels, or as mean and standard deviations. To determine gender disparities among all residents independent *t* tests were performed, whereas variance analysis was used to analyse gender-related difference between male and female residents independent of their country of origin. In case of missing normal distribution, parameter free alternatives (Mann–Whitney *U* tests, Kruskal–Wallis tests) were chosen. The questions on whether gender impacts on a feeling of security during standard interventions and surgeries, as well as on overall confidence in terms of being prepared for work as a specialist, were answered with the help of multivariate linear regression models. Based on the available literature, the following covariates have been included in the model and tested with a forward selection strategy: year of training, country, workload (full-time versus part-time), receiving regular feedback, and implemented simulation training at the clinic [9, 11, 15]. A two-sided *p* value < 0.05 was considered to be statistically significant.

## Results

### Study population

**Fig. 1:**
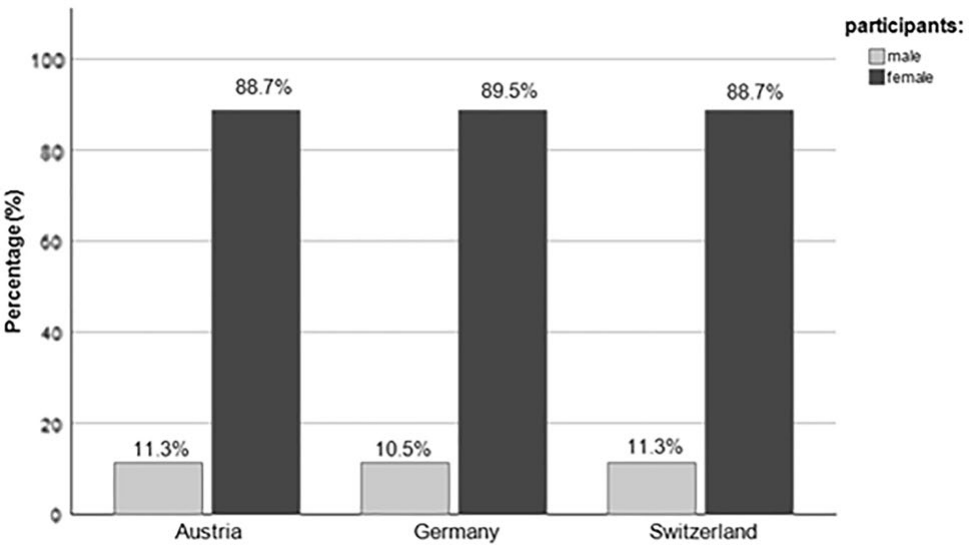
Gender distribution in the countries Austria, Germany, and Switzerland

422 participants took part in the survey, of which 375 (88.9%) were female, 46 (10.9%) were male, and one (0.2%) was divers. The diverse participant was excluded from further analyses. The gender distribution was comparable in all three countries (Fig. 1).

The median age of the participants was 32 (22–55) years in the total cohort. Participants had graduated from medical university in 2015 (1990–2019), had been in training for 3 (0–10) years, and had been working at their current training institution for 23 months (0–190 months). 29.9% of the participants were working at an institution with up to 200 beds, 28.7% at an institution with between 201 and 499 beds, and 41.4% at an institution with more than 500 beds. Baseline characteristics stratified by gender and country are shown in Table 1.

**Table 1:** Baseline characteristics of the study cohort stratified by gender and country

	Variables median (min–max)								Frequency (proportion, %)		
	Age (years)		Year of graduation		Years of training (irrespective of workload)		Employment at current training institution (months)		Number of beds at current workplace		
Switzerland	Males (*N* = 13)	Females (*N = *102)	Males (*N* = 13)	Females (*N* = 102)	Males (*N* = 13)	Females (*N* = 102)	Males (*N* = 13)	Females (*N* = 102)		Males (*N* = 13)	Females (*N* = 102)
	35 (26–47)	32 (26–48)	2013 (2004–2017)	2014 (2001–2018)	3.5 (2–6)	4 (1–10)	15 (3–84)	20 (1–84)	< 200 beds	4 (30.8%)	39 (38.2%)
									201–499 beds	3 (23%)	25 (24.5%)
									≥ 500 beds	6 (46.2%)	38 (37.3%)
Germany	Males (*N* = 22)	Females (*N* = 187)	Males (*N* = 22)	Females (*N* = 187)	Males (*N* = 22)	Females (*N* = 187)	Males (*N* = 22)	Females (*N* = 187)		Males (*N* = 22)	Females (*N* = 187)
	32 (26–55)	31 (22–44)	2016 (1990–2019)	2016 (2002–2018)	3 (1–6)	3 (0–9)	33 (4–84)	24 (0–190)	< 200 beds	3 (13.6%)	42 (22.5%)
									201–499 beds	7 (31.8%)	58 (31%)
									≥ 500 beds	12 (54.6%)	87 (46.5%)
Austria	Males (*N* = 11)	Females (*N* = 86)	Males (*N* = 11)	Females (*N* = 86)	Males (*N* = 11)	Females (*N* = 86)	Males (*N* = 11)	Females (*N* = 86)		Males (*N* = 11)	Females (*N* = 186)
	31 (27–54)	31 (25–40)	2016 (1997–2018)	2014 (2008–2019)	3 (2–3)	4 (0–10)	20 (3–120)	25.5 (1–96)	< 200 beds	2 (18.2%)	36 (41.9%)
									201–499 beds	5 (45.4%)	23 (26.7%)
									≥ 500 beds	4 (36.4%)	27 (31.4%)

### Co-factors (working time, mentoring/feedback, simulation training) that may influence the quality of training programs

There was a significant gender-related difference in working time models in the total cohort. Of the 375 female doctors, 74.4% were working fulltime, whereas 95.7% of the men worked 100% of the standard working week (*p* = 0.002). Among the other female participants, 12.5% were working between 80 and 95%, 11.2% were working 60–75%, and 1.9% were working 40–55%. Conversely, 4.3% of the male participants worked 40–55%; other working time models were not represented among them. In more detail, women worked 50 h per week on average, whereas it was noted that men worked 55 h per week (*p* = 0.027).

During their training, 37.3% of the female doctors felt strongly that they received regular feedback on their practical skills, whereas 30.5% of the men felt strongly that they did (*p* = 0.277). The majority of the participants—73.1% of the women and 78.3% of the men—found a supervisor who they could contact with questions regarding their education (*p* = 0.188). Furthermore, 53.3% of the female and 67.4% of the male participants stated that they had a mentor with whom they could discuss their carer (*p* = 0.106).

Concerning simulation trainings for obstetric procedures, 51.5% of the women and 56.5% of the men responded that these are not implemented at their hospitals (*p* = 0.973). Similarly, 75.2% of the female doctors and 69.6% of the male doctors stated that their clinics do not offer simulation trainings for gynaecological interventions (*p* = 0.519). When trainings were implemented, 21.1% of the women and 23.9% of the men managed to take part within designated time periods (*p* = 0.447). In more detail, Table 2 summarizes the variables working time models, receiving mentoring/feedback, and implementation of simulation trainings stratified by gender and country.

**Table 2:** Variables working time models, receiving mentoring/feedback, and implementation of simulation trainings stratified by gender and country

Variables frequency (proportion, %)	Switzerland		Germany		Austria	
	Males (*N* = 13)	Females (*N* = 102)	Males (*N* = 22)	Females (*N* = 187)	Males (*N* = 11)	Females (*N* = 86)
Working-time models						
100%	13 (100%)	72 (70.6%)	21 (95.5%)	132 (70.6%)	10 (90.9%)	75 (87.2%)
80–95%	0 (0%)	14 (13.7%)	0 (0%)	33 (17.6%)	0 (0%)	0 (0%)
60–75%	0 (0%)	13 (12.8%)	0 (0%)	20 (10.7%)	0 (0%)	9 (10.5%)
40–55%	0 (0%)	3 (2.9%)	1 (4.5%)	2 (1.1%)	1 (9.1%)	2 (2.3%)
< 40%	0 (0%)	0 (0%)	0 (0%)	0 (0%)	0 (0%)	0 (0%)
Receiving mentoring/feedback						
Receiving regular feedback on their practical skills						
Strongly agree	4 (30.8%)	50 (49%)	5 (22.7%)	66 (35.3%)	5 (45.5%)	24 (27.9%)
Having a supervisor to contact regarding education						
Yes	10 (76.9%)	88 (86.3%)	16 (72.7%)	123 (65.8%)	10 (90.9%)	63 (73.3%)
No	3 (23.1%)	14 (13.7%)	6 (27.3%)	64 (34.2%)	1 (9.1%)	23 (26.7%)
Having a supervisor to contact regarding career						
Yes	10 (76.9%)	73 (71.6%)	12 (54.5%)	88 (47.1%)	9 (81.8%)	39 (45.3%)
No	3 (23.1%)	29 (28.4%)	10 (45.5%)	99 (52.9%)	2 (18.2%)	47 (54.7%)
Implementation of simulation trainings						
Simulation trainings for obstetric procedures						
Yes	7 (53.8%)	65 (63.7%)	5 (22.7%)	55 (29.4%)	6 (54.5%)	47 (54.7%)
No	6 (46.2%)	37 (36.3%)	16 (72.7%)	123 (65.8%)	4 (36.4%)	36 (41.8%)
I do not know	0 (0%)	0 (0%)	1 (4.6%)	9 (4.8%)	1 (9.1%)	3 (3.5%)
Simulation trainings for gynaecological interventions						
Yes	4 (30.8%)	29 (28.4%)	4 (18.2%)	31 (16.6)	2 (18.2%)	15 (17.4%)
No	9 (69.2%)	66 (64.7%)	16 (72.7%)	148 (79.1%)	7 (63.6%)	68 (79.1%)
I do not know	0 (0%)	7 (6.9%)	2 (9.1%)	8 (4.3%)	2 (18.2%)	3 (3.5%)
Participation in designated time periods						
Yes	5 (38.5%)	44 (43.1%)	1 (4.5%)	18 (9.6%)	5 (45.5%)	17 (19.8%)
No	2 (15.4%)	27 (26.5%)	5 (22.7%)	50 (26.7%)	1 (9.1%)	26 (30.2%)
I do not know	0 (0%)	2 (2%)	1 (4.5%)	1 (0.5%)	1 (9.1%)	4 (4.7%)
Missing data	6 (46.1%)	29 (28.4%)	15 (68.3%)	118 (63.2%)	4 (36.3%)	39 (45.3%)

### Confidence in performing standard OBGYN procedures

The feeling of confidence in performing standard gynaecological surgeries, including curettage, hysteroscopy, and simple laparoscopy, was estimated in the total cohort by females at 5.10 (± 1.47) and by males at 5.53 (± 1.46) points using a scale reaching from 1 (“not confident at all”) to 7 (“very confident”). Concerning standard obstetric procedures, including forceps delivery, vacuum delivery, postpartum bleeding management, shoulder dystocia, breech position delivery, and caesarean section, overall confidence was rated by female doctors at 3.47 (± 1.01) and by male doctors at 3.91 (± 1.23). Both findings show a significant gender difference, with women being less confident than men (*p* = 0.033; *p* = 0.007). Table 3 presents in more detail confidence ratings for each gynaecological and obstetric procedure for the total cohort as well as stratified by gender and country.

**Table 3:** Confidence ratings, reaching from 1 (“not confident at all”) to 7 (“very confident”), of standard gynaecological and obstetrical procedures stratified by gender and country

Confidence ratings (mean ± SD)	Total			Switzerland			Germany			Austria		
	Females (*N* = 375)	Males (*N* = 46)	*p* value	Females (*N* = 102)	Males (*N* = 13)	*p* value	Females (*N* = 187)	Males (*N* = 22)	*p* value	Females (*N* = 86)	Males (*N* = 11)	*p* value
Obstetric procedures												
Forceps delivery	1.15 (± 0.60)	1.39 (± 1.09)	0.024	1.18 (± 0.72)	1.54 (± 0.97)	0.027	1.15 (± 0.58)	1.50 (± 1.37)	0.151	1.12 (± 0.58)	1.00 (± 0.0)	0.468
Vacuum delivery	3.61 (± 1.84)	4.35 (± 2.08)	0.011	4.43 (± 1.66)	5.77 (± 1.3)	0.005	3.11 (± 1.82)	3.64 (± 2.17)	0.267	3.71 (± 1.76)	4.09 (± 1.92)	0.626
Postpartum bleeding	4.81 (± 1.43)	4.98 (± 1.54)	0.458	5.12 (± 1.21)	5.92 (± 1.04)	0.016	4.76 (± 1.47)	4.73 (± 1.49)	0.775	4.56 (± 1.55)	4.36 (± 1.75)	0.613
Shoulder dystocia	3.86 (± 1.50)	4.46 (± 1.83)	0.014	4.19 (± 1.41)	5.62 (± 1.26)	0.002	3.72 (± 1.5)	4.09 (± 1.77)	0.455	3.97 (± 1.58)	4.82 (± 2.04)	0.912
Breech position	1.87 (± 1.24)	2.41 (± 1.51)	0.006	2.11 (± 1.33)	2.69 (± 1.6)	0.148	1.91 (± 1.27)	2.00 (± 1.45)	0.747	1.49 (± 0.97)	2.91 (± 1.45)	< 0.001
Gynaecological procedures												
Suction curettage	5.92 (± 1.37)	6.09 (± 1.24)	0.440	6.22 (± 1.11)	6.62 (± 0.87)	0.067	5.84 (± 1.38)	5.86 (± 1.39)	0.799	5.74 (± 1.59)	5.91 (± 1.22)	0.990
Hysteroscopy	5.39 (± 1.66)	5.61 (± 1.72)	0.400	5.62 (± 1.43)	6.38 (± 0.96)	0.033	5.19 (± 1.75)	5.05 (± 1.89)	0.792	5.55 (± 1.68)	5.82 (± 1.78)	0.522
Simple laparoscopy	4.00 (± 1.86)	4.89 (± 1.80)	0.002	4.48 (± 1.63)	5.77 (± 1.3)	0.007	3.87 (± 1.88)	4.36 (± 1.91)	0.296	3.72 (± 1.99)	4.91 (± 1.81)	0.064
Primary caesarean section	5.53 (± 1.55)	5.87 (± 1.52)	0.160	5.60 (± 1.26)	6.31 (± 1.03)	0.037	5.47 (± 1.71)	5.68 (± 1.62)	0.519	5.58 (± 1.5)	5.73 (± 1.79)	0.539

Multiple regression analysis (*F*[5,415] = 4.97; *p* < 0.001; *n* = 421) showed that gender (*p* = 0.03), year of training (*p* < 0.001), receiving regular feedback (*p* = 0.01), and implementation of simulation training (*p* = 0.002) are independent variables and have a significant effect on confidence level when it comes to performing standard gyneacological procedures (Table 4A). Being a woman significantly decreases the feeling of confidence (odds ratio [OR] − 1.53; 95% confidence interval [CI] − 2.84 to − 0.22; *p* = 0.03). Regarding confidence concerning standard obstetric interventions, multiple regression analysis (*F*[5,415]  = 8.86; *p* < 0.001; *n* = 421) revealed that gender (*p* < 0.001), workload (*p* = 0.04), year of training (*p* < 0.001), and implementation of simulation training (*p* < 0.001) have a significant effect (Table 4B). Thus, being a woman significantly lowers confidence level (OR − 3.43; CI − 5.28 to − 1.58; *p* < 0.001).

**Table 4:** Multivariate regression model of variables influencing the confidence level of performing standard OBGYN procedures

	OR	95% CI	*p* value
A. Standard gyneacological procedures			
Gender			
Male	Reference		0.03
Female	− 1.53	− 2.84 to − 0.22	
Year of training			
0–3 years	Reference		< 0.001
> 3 years	1.97	1.13 to 2.82	
Country			
Non-Austria	Reference		0.37
Austria	− 0.04	− 0.04 to 0.10	
Workload			
Full-time	Reference		0.41
Part-time	− 0.40	− 0.40 to 0.10	
Receiving regular feedback			
No	Reference		0.01
Yes	1.23	0.41 to 2.10	
Implementation of simulation trainings			
No	Reference		0.002
Yes	1.10	0.07 to 2.19	
B. Standard obstetric interventions			
Gender			
Male	Reference		< 0.001
Female	− 3.43	− 5.28 to − 1.58	
Year of training			
0–3 years	Reference		< 0.001
> 3 years	2.27	1.09 to 3.45	
Country			
Non-Austria	Reference		0.07
Austria	− 0.09	− 0.09 to 0.99	
Workload			
Full-time	Reference		0.04
Part-time	− 1.53	− 2.89 to − 0.17	
Receiving regular feedback			
No	Reference		0.05
Yes	1.18	0.02 to 2.33	
Implementation of simulation trainings			
No	Reference		< 0.001
Yes	2.72	1.56 to 3.88	

### Confidence in being prepared for working as a specialist

**Fig. 2:**
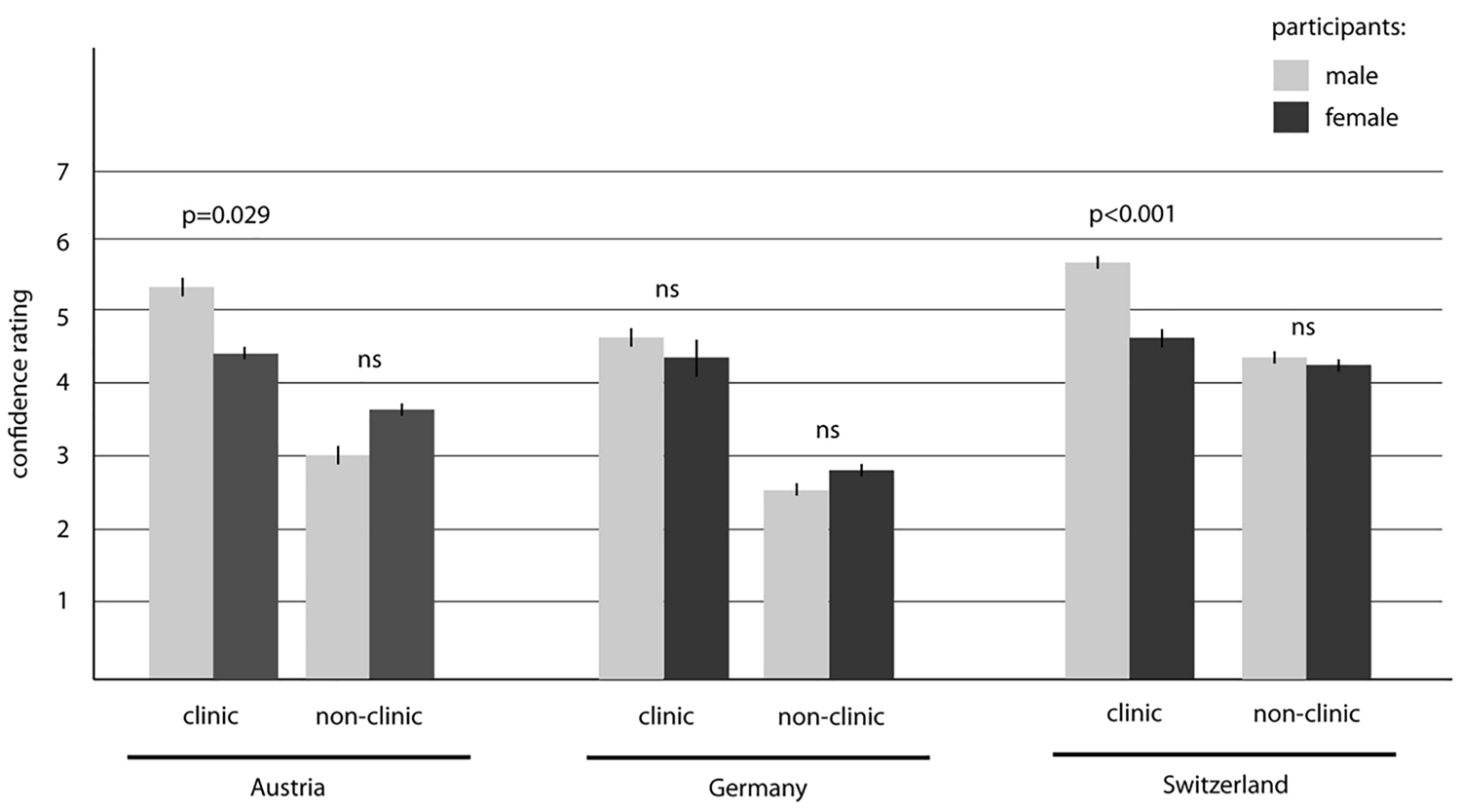
Feeling of confidence in being prepared for working as a specialist stratified by gender and country. *ns*: non significant

The evaluation concerning the feeling of confidence in being prepared for working as a specialist in a clinic in the total cohort presented a mean value of 5.02 (± 1.48) for men and 4.36 (± 1.43) for women; this difference turned out to be significant (*p* = 0.003). The confidence ratings, however, concerning working as specialist in an outpatient setting was 3.20 (± 1.73) for male doctors and 3.43 (± 1.61) for female doctors—a non-significant finding (*p* = 0.353). Both analyses, stratified by gender and country, are shown in Fig. 2.

Multivariate regression analysis (*F*[5,415] = 11.38; *p* < 0.001; *n* = 421) showed that gender (*p* < 0.001), receiving regular feedback (*p* < 0.001), year of training (*p* < 0.001), and the implementation of simulation training (*p* < 0.001) impact the feeling of security in terms of working as a specialist in a hospital (Table 5A), whereas only the last two variables have a significant effect (both *p* < 0.001) on feeling prepared for working in an outpatient practice (Table 5B). The “female factor” significantly decreases the self-confidence of working in a hospital.

**Table 5:** Multivariate regression model of variables influencing the feeling of security in terms of working as a specialist

	OR	95% CI	*p* value
A. Feeling of security in terms of working as a specialist in a hospital			
Gender			
Male	Reference		*p* < 0.001
Female	− 0.74	− 1.16 to − 0.33	
Year of training			
0–3 years	Reference		0.004
> 3 years	0.36	0.13 to 0.67	
Country			
Non-Austria	Reference		0.26
Austria	− 0.05	− 0.06 to 0.99	
Workload			
Full-time	Reference		0.39
Part-time	− 0.04	− 0.04 to 0.99	
Receiving regular feedback			
No	Reference		< 0.001
Yes	0.48	0.21 to 0.74	
Implementation of simulation trainings			
No	Reference		< 0.001
Yes	0.59	0.32 to 0.87	
B. Feeling of security in terms of working as a specialist in an outpatient setting			
Gender			
Male	Reference		0.37
Female	0.04	0.04 to 0.99	
Year of training			
0–3 years	Reference		< 0.001
> 3 years	0.52	0.21 to 0.83	
Country			
Non-Austria	Reference		0.37
Austria	0.04	0.04 to 0.99	
Workload			
Full-time	Reference		0.26
Part-time	− 0.05	− 0.06 to 0.10	
Receiving regular feedback			
No	Reference		0.07
Yes	0.08	0.09 to 0.97	
Implementation of simulation trainings			
No	Reference		< 0.001
Yes	0.78	0.48 to 1.09	

## Discussion

### Main findings

The phenomenon of the “feminization of medicine” exists in the field of OBGYN. Our study, therefore, analysed, for the first time, gender-related differences in the self-confidence of practical and surgical skills among OBGYN trainees in the context of year of training, country, workload (full-time versus part-time), reception of feedback, and implementation of simulation training—variables known from literature to potentially interfere with the quality of training programs [9, 11, 15]. Intriguingly, our data identified, after adjustment for these confounders, that gender significantly affects the confidence of doctors in terms of performing both obstetric and gynaecological standard procedures, with women being less secure than men. Furthermore, women feel less prepared, through their training in working as specialist in a hospital setting, than their male colleagues.

### Strengths and limitations

Although our study is the first to highlight that being female negatively impacts the confidence of practical and surgical skills among OBGYN residents, there are also some suggestions for enhancing the study design for future surveys covering this topic. Collecting more detailed information about the number of children and the distribution of childcare would enlarge statistical analyses and conclusions. The median age of our participants was 32 (women) and 33 (men), which meant that family planning was probably an actual issue for most of them. In our analysis we included the variables “year of training” and “workload” (full-time versus part-time), to approximate different training levels of the residents. Future studies could also directly ask for the exact time (in hours) spent in the operating room. Finally, having participants assess their self-esteem would also significantly improve the quality of interpretation.

### Interpretation

To date, the available literature suggests some framework conditions that potentially interfere with training quality, such as work-time models [9]. Our data clearly show the distribution of work-time models between male and female OBGYN doctors, with men working almost exclusively full-time and women working part-time (< 95%) significantly more frequently. In accordance with our findings, studies confirm that working part-time (< 95%) is considered to be more attractive to female doctors, while working full-time is considered to be more attractive to male doctors [16]. Nevertheless, Lermann et al. found that 60% of physicians in training in OBGYN were dissatisfied with their current job situation, citing irregular and long working hours as some of the main reasons, irrespective of their chosen working time model [17, 18]. Schott et al. showed that part-time employees were also working night shifts and weekends and that the number of monthly night shifts did not differ between full-time employees and part-time employees, at > 75% [18]. This suggests that, even with the option of working part-time, female doctors´ care duties, such as caring for a child or an elderly relative, might not be very compatible with the working situation at some workplaces. In our study, working hours differed on average by 5 h between female and male doctors. Overall, this plays a part in women facing structural obstacles when pursuing both family and an academic career. For the latter, female physicians must conduct research and train new doctors—besides to their duties of seeing, diagnosing and treating patients. This triad can often not be managed within the regular working hours. In this context, studies have shown that women in higher positions have fewer children than their male counterparts and that they are less likely to be primarily responsible for childcare [8]. Similarly, our data reveal that working part-time negatively interferes with training progress, in the sense that female doctors feel less secure in performing standard gynaecological and obstetric procedures.

In terms of mentoring and receiving feedback, no gender-related differences were found in our cohort. Conversely, Stamm et al. identified that up to 50% of doctors in postgraduate training had a mentor, with a significant gender gap, where more men had a mentor than their female counterparts [11]. The authors clearly demonstrated that having a mentor and career support positively predicted both subjective and objective career success [11]. Similarly, our data showed that receiving feedback on a regular basis concerning practical and surgical skills positively influenced the feeling of confidence in performing standard procedures and the feeling of being well-prepared for working as specialist in a hospital. Since Riepen et al. found that up to 62.4% of female OBGYN doctors have doubts about their ability to succeed in an academic career, as opposed to 7.7% of men, the equal distribution of mentoring among male and female doctors is highly warranted, especially for more senior OBGYN positions, which are still mostly held by male doctors [19].

Furthermore, the body of literature has proven that simulation training is an important tool for both improving and evaluating technical and practical skills, especially in laparoscopic surgery [15, 20]. For the field of OBGYN, Winder et al. have recently demonstrated that residents who had the opportunity to complete simulation trainings regularly felt safer performing standard gynaecological and obstetric procedures compared with colleagues who had not had such an opportunity [15]. Notably, it was shown by Mannella et al. that the judgment expressed by external observers corresponded to the trainee’s perceptions concerning their skills in simulation training [20]. In general, the intrinsic feeling of safety is a significant parameter mirroring the quality of a training program [20].

Therefore, the present study aimed to assess gender-related differences in surgical and practical skills by evaluating the feeling of confidence in performing standard gynaecological (curettage, hysteroscopy, and simple laparoscopy) and obstetric (forceps delivery, vacuum delivery, postpartum bleeding management, shoulder dystocia, breech position delivery and caesarean sections) procedures. Intriguingly, the data found that gender independently impacts on the practical and surgical skills in performing most procedures, with women being less confident than their male counterparts. Furthermore, the study identified that female doctors feel significantly less prepared to work as specialist in a hospital. Potential covariates including year of training, country, workload, receiving regular feedback, and implemented simulation training had been considered within statistical models. Since the medical activity profile is different between a hospital and an outpatient-setting, with more surgeries and emergency-cases in the first mentioned condition, it is not surprising that the gender-related discrepancies found in the feeling of security in working as a specialist only occurred in the setting of hospital-employment.

The most important question that emerges from our data is why female physicians feel less confident and insecure while working in their OBGYN discipline. As self-esteem is one of the most widely studied constructs in the social science, Manne-Goehler et al. was one of the first studies that explored the relationship among self-esteem, gender, and career outcomes in academic medicine [21–23]. Although the author demonstrated that female doctors generally have a lower self-esteem compared to their male counterparts, the magnitude of the difference was small; making them argue that this construct seems not to be the missing mediator between gender and professional success [21]. Another psychological construct to explain the found gender-discrepancies may be self-efficacy, the feeling about the own ability to function in different situations [24]. Flyckt et al. demonstrated that female surgeons under-estimate their surgical abilities in a laparoscopic simulation training, although they showed the same performance as male surgeons [24].This difference in self-efficacy, or “confidence gap,” between male and female trainees may also be a reason why female physicians significantly less likely pursue fellowship trainings in surgical disciplines than their male counterparts [24, 25]. In this context, emerging research showed that female physicians significantly struggle more often with the “impostor syndrome”, meaning that they feel they do not deserve their success, than their male counterparts [26]. To summarize, a multicausal interaction of diverse structural and psychological factors resulting in female OBGYN residents feeling less confident and insecure in performing standard gynaecological and obstetric procedures and to work as a specialist are likely.

Our findings have implications for resident training and indicate potential for improvement. Besides the removal of structural obstacles, ways of increasing practical/surgical confidence of female OBGYN residents must be prioritized to allow for optimal education and growth in resident trainees. For example, the opportunity to perform additional cases with a single surgical same-sex mentor to build surgical volume and feelings of competence, would enhance the feeling of self-efficacy in female physicians who underestimate their abilities [24]. In addition, the offer of regular coaching lessons would help to enhance the building of a strong female “surgical personality [24]”.

However, awareness of gender career bias must also be raised within the local training environment as an important first step toward minimizing effects hindering the professional development of female surgeons [27]. Recently, Salle et al. demonstrated that both implicit and explicit gender bias exist among healthcare workers across all social categories (gender, race, title, region of the United States, and country of origin) [27]. The author found that even male and female surgeons associated men with career and surgery and women with family and family medicine [27].

However, research also identified gender bias disadvantaging male physicians [28–30]. Emerging evidence show that a strong preference for same-gender physicians is expressed by 20–45% of female patients, especially when it comes to gender-sensitive examinations [31, 32]. This pattern was found across different countries worldwide [29, 31]. Studies examining clerkship experiences even noted men disproportionately reporting exclusion from clinical experiences in OBGYN [28]. Beyond patient gender bias, preconceived gender biases held by male physicians may contribute to the declining proportion of male OBGYN residents.

Given the great diversity of patients, leaders of OBGYN must ensure that the employment of physicians with different gender, ethnicity, and social backgrounds is prioritized. Integration of divers OBGYN specialists not only within clinical practice, but also in leadership positions will increase an organizational success and the availability of divers role models and potential mentors for all applicants and residents [27].

## Conclusion

The number of women entering medicine, especially OBGYN, is steadily increasing, thereby forcing hospitals to reflect on current structures and to adapt to the changing demands of future doctors. Based on our data, which revealed that female doctors feel less secure in performing standard gynaecological and obstetric interventions and in being prepared to work as a specialist, improvements of residency programs are highly warranted. Targeted promotion for female doctors to overcome factors (e.g., structural and/or psychological factors) depressing their overall confidence must be implemented.

## Data Availability

The datasets generated during the current study are not publicly available. Appropriate forms of data sharing can be arranged after a reasonable request to the corresponding author.
